# Impaired DNA Double-Strand Break Repair in Irradiated Sheep Lung Fibroblasts: Late Effects of Previous Irradiation of the Spinal Thecal Sac

**DOI:** 10.3390/cancers16172968

**Published:** 2024-08-26

**Authors:** Bassem Youssef, Charbel Feghaly, Joelle Al Choboq, Jolie Bou-Gharios, Rafka Challita, Joyce Azzi, Hanine Bou Hadir, Fabienne Abi Antoun, Tarek Araji, Phillip J. Taddei, Fady Geara, Pierre Sfeir, Abdo Jurjus, Wassim Abou-Kheir, Larry Bodgi

**Affiliations:** 1 Department of Radiation Oncology, American University of Beirut Medical Center, Beirut 1107 2020, Lebanon; by04@aub.edu.lb (B.Y.); cf35@aub.edu.lb (C.F.); joelle.al-choboq@inserm.fr (J.A.C.); j.bou-gharios@icans.eu (J.B.-G.); rsc13@mail.aub.edu (R.C.); joyce.a.azzi@gmail.com (J.A.); hb106@aub.edu.lb (H.B.H.); fabienneabiantoun@gmail.com (F.A.A.); philtaddei@proton.me (P.J.T.); gearaf@clevelandclinicabudhabi.ae (F.G.); 2 Department of Anatomy, Cell Biology and Physiological Sciences, Faculty of Medicine, American University of Beirut, Beirut 1107 2020, Lebanonaj00@aub.edu.lb (A.J.); wa12@aub.edu.lb (W.A.-K.); 3 Department of Radiation Oncology, Texas Oncology, Dallas, TX 75251, USA; 4 Department of Surgery, American University of Beirut Medical Center, Beirut 1107 2020, Lebanon; ps04@aub.edu.lb; 5 U1296 Unit, “Radiation: Defense, Health and Environment”, Centre Léon-Bérard, Inserm, 28 Rue Laennec, 69008 Lyon, France

**Keywords:** pediatric cancer, radiosensitivity, radiotherapy, DNA double-strand breaks, DNA repair

## Abstract

**Simple Summary:**

Childhood cancer survivors treated with radiotherapy face the likelihood of long-term complications, including mutations. In order to assess the long-term effect of radiotherapy on the capacity of cells to repair their DNA double-strand breaks (DSBs), five lambs received medulloblastoma radiotherapy to the thecal sac, with three lambs serving as controls. Four years later, lung biopsies were taken and fibroblast cells were amplified and re-irradiated. The cells from the previously treated sheep showed a significant impairment of their DNA DSB repair mechanism, highlighting a potential increase in radiosensitivity. Our results show that previous irradiation can impair the DNA DSB repair mechanism of ovine lung fibroblasts.

**Abstract:**

Children with cancer previously treated with radiotherapy face the likelihood of side effects that can be debilitating or fatal. This study aimed to assess the long-term effect of medulloblastoma radiotherapy on the DNA double-strand break (DSB) repair capability of primary fibroblasts derived from lung biopsies of previously irradiated young sheep. This study included biopsies from three control and five irradiated sheep. The treated sheep had previously received spinal radiotherapy at a total dose of 28 Gy, which is equivalent to pediatric medulloblastoma treatment. Lung biopsies were taken 4 years post-irradiation from high-dose (HD, >18 Gy) and low-dose (LD, <2 Gy) regions. Fifteen cell lines were extracted (six control, four LD and five HD). The cells were irradiated, and DNA DSB repair was analyzed by immunofluorescence. Clonogenic, trypan blue and micronuclei assays were performed. Both the HD and LD cell lines had a significantly higher number of residual γH2AX foci 24 h and a significant decrease in pATM activity post-irradiation compared to the control. There was no statistically significant difference in the clonogenic assay, trypan blue and micronuclei results. Our study showed that a previous irradiation can impair the DNA DSB repair mechanism of ovine lung fibroblasts.

## 1. Introduction

It is common and often necessary to supplement surgery and chemotherapy of children with medulloblastoma (MB) with craniospinal irradiation (CSI) [1,2]. Unavoidable in-field, partially in-field, and out-of-field (i.e., peripheral) radiation can induce mutations in normal tissues, resulting in a risk of late effects, including cancer [3]. While radiotherapy (RT) is planned to target existing cancerous cells, surrounding tissues and organs, such as the lungs, bones and heart, receive a non-negligible dose during treatment. This disadvantageous radiation dose can induce a wide range of DNA damage, such as base damage, single-strand breaks and double-strand breaks (DSBs), with the latter being lethal and the most difficult to repair [4]. Any failure to repair DNA DSBs can lead to radio-induced toxicities in the case of unrepaired damage, or mutations if misrepaired. In the long term, such mutations can lead to the inactivation of tumor suppressor genes and/or activation of oncogenes, hence the increased risk of developing a second cancer. Mutations can also target DNA repair proteins, therefore increasing both the radiosensitivity and cancer risk [5,6,7]. However, there are no studies that have focused on the long-term effect of ionizing radiation at the molecular and cellular levels, and more specifically on the capacity of the cells to repair their DSBs.

In response to genotoxic stress, the cell will activate DNA damage repair pathways. The non-homologous end-joining repair pathway is the main repair pathway for cells in G0/G1. Studies have shown that ataxia telangiectasia mutated (ATM), a major DSB repair protein located in the cytoplasm, autophosphorylates and becomes monomerized before transiting to the nucleus via nucleoshuttling [4,8,9]. The nuclear forms of pATM will phosphorylate the histone H2AX (γH2AX), which will trigger the DSB repair pathway. When assessed by immunofluorescence (IF), the kinetics of both pATM and γH2AX were shown to be reliable predictors of normal tissue radiosensitivity [9,10,11,12,13]. Any delay in ATM nucleoshuttling and any decrease in the capacity of the cells to repair their radio-induced DNA DSBs is correlated with a higher radiosensitivity.

Biopsies of lung cells were taken from sheep previously treated with spinal fields characteristic of CSI. Healthy lambs were chosen as animal models for young children, and the follow-up period was 4 years. Primary fibroblasts were derived from these cells, amplified, and re-irradiated with a single dose of 2 Gy in order to characterize their molecular and cellular radiosensitivity.

Our model mimics the treatment of children with MB. Biopsies were taken from the sheep lungs upon adulthood to assess the different types of variations in response to oxidative stress. We focused on the lungs because they are considered to be one of the main organs at risks, and also because of the advantage of some parts of the lungs receiving as high as 70% of the prescribed dose (in-field) as well as the low, out-of-field dose.

## 2. Materials and Methods

### 2.1. The Sheep Treatment

Healthy lambs of the Awassi breed were collected from farms in the Beqaa Valley in Lebanon, and lived in the farms of the Advancing Research Enabling Communities center (AREC) in the Beqaa region of Lebanon. The irradiation took place at the American University of Beirut Medical Center (AUBMC) when the lambs were aged between 3 and 5 weeks. Experiments were performed under the supervision of the Institutional Animal Care and Use Committee (IACUC) of the American University of Beirut (AUB).

A total of 8 sheep were included in this study: 3 untreated controls (2 females and 1 male), and 5 treated (3 females and 2 males).

The animals were transported to the treatment facility under various levels of sedation (ketamine 10 mg/Kg) and periodically re-sedated until the procedures were complete. The animals were shaved along the spine prior to imaging, and their spines were marked at midline with a permanent marker along the positioning lasers. For each sheep, a computed tomography (CT) image set was reconstructed with 3 mm slices, and the images were imported to a treatment planning system.

The irradiation of the spine was performed in a total of 8 fractions, twice per week, with a dose of 3.38 Gy per fraction. Based on the studies showing that sheep radiosensitivity is comparable to that of humans, in addition to the fact that the bone structure is comparable, we applied a general late-effect alpha/beta ratio of 3 Gy to adjust the dose and fraction to two sessions per week [14,15]

This is biologically equivalent to the total dose of 36 GyEq delivered in 20 sessions, which is considered the standard dose prescribed for the spine in patients with high-risk MB [16,17]. The sheep were positioned for treatment using lasers and cone beam computed tomography. Treatment was performed using 6 MV photon fields to deliver the prescribed dose.

**Figure 1:**
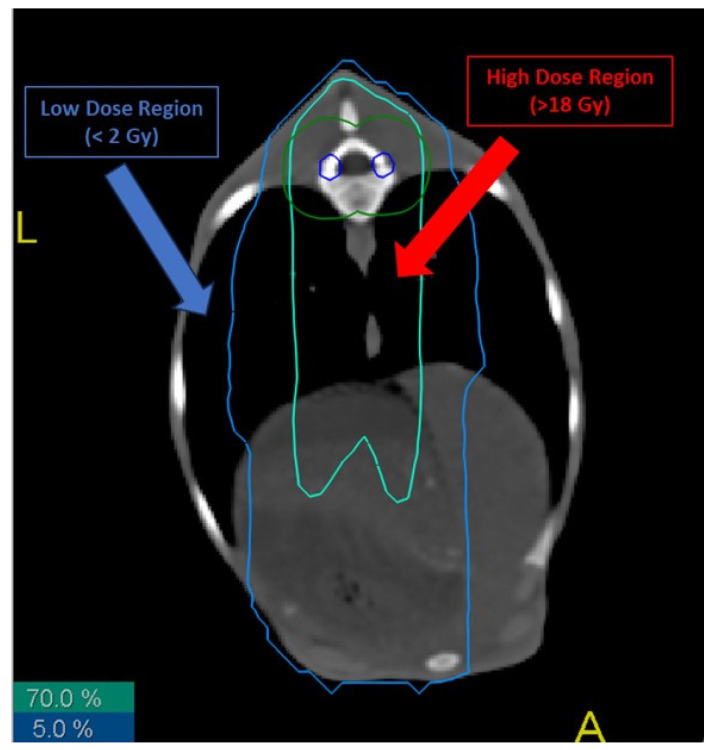
Different radiation isodoses in the lungs of treated sheep. Biopsies from high- and low-dose regions were taken, cultured and analyzed. Blue line represents the 5% isodose of the total received dose, while red line represents the 70% isodose.

We defined two irradiation regions (Figure 1):

- Low-dose (LD) region: area of the sheep that received a total dose lower than 2 Gy.
- High-dose (HD) region: area of the sheep that received a total dose higher than 70% of the total dose to the thecal sac, i.e., more than 18 Gy.

### 2.2. Sheep Euthanasia and Lung Tissue Sampling

Sheep were euthanized 4 years after their treatments in 3 batches, two weeks apart. All procedures were performed under the supervision of the Institutional Animal Care and Use Committee (IACUC) of the AUB and a specialized cardiothoracic surgeon. One day before the simulation imaging procedure, the selected sheep were shaved, washed, and prepared. The sheep were euthanized under anesthesia. A cardiothoracic surgeon performed the surgeries and took the biopsies from the specified locations.

This study consisted of analyzing cells from tissue in two regions in the lung of each sheep, LD and HD. Samples were taken from treated and untreated sheep, with the untreated sheep cells serving as a baseline for analysis (D = 0 Gy). A total of 16 samples were collected (6 control, 5 LD and 5 HD). Absorbed dose in each of these were determined during treatment planning (Figure 1). Excised biopsies were placed in sterile tubes containing complete media (DMEM-F12 HAM supplemented with 0.2% plasmocin prophylactic, 0.2% gentamicin amphotericin B, 1% penicillin streptomycin and 10% FBS).

### 2.3. Primary Cell Culture

The samples were cut into very small pieces using sterile blades and forceps on the lid of bacterial dishes, transferred into a conical flask containing collagenase II (5 mg/mL) and then incubated in a 5% CO_2_ humidified incubator at 37 °C overnight. Tubes were then centrifuged, and the supernatant was removed. The pellet was washed with complete DMEM-F12 HAM, and the supernatant was removed after centrifugation. Samples were then incubated with Trypsin (1 mL) for 20 min. After 20 min, tissues were dissociated by pipetting; they were then centrifuged and after this, the supernatant was removed and tissues were resuspended with 1 mL complete media. A 40 µm cell strainer was used to strain the cells. A volume of 1 mL working solution of red blood cell lysis solution was added to all tubes for 15 min at room temperature in order to lyse red blood cells. After centrifugation, the pellet was resuspended with 1 or 2 mL complete media (depending on the pellet size). Cells were counted using trypan blue (50 µL cells + 5 µL trypan blue). A total of 250,000 cells/well were taken for culture and the media were changed every other day.

After handling the cells, we were able to establish 15 cell lines (6 control, 4 LD and 5 HD), all of which were included in this study. The cell lines were as follows:

Control: C1LL, C1LH, C2LL, C2LH, C3LL, C3LH.

LD: X2LL, X3LL, X4LL, X5LL.

HD: X1LH, X2LH, X3LH, X4LH, X5LH.

### 2.4. Cell Line Irradiation

Irradiations of cells were performed on the XRAD 225kV (Precision XRAY, Madison, CT, USA) research irradiator at AUB with a dose rate of 3 Gy.min^−1^.

### 2.5. Trypan Blue Exclusion Assay

The trypan blue exclusion assay was used to determine the number of living cells in a cell suspension.

Cells were seeded in 24-well plates and incubated in a humidified incubator at 37 °C and 5% CO_2_. At 24, 48 and 72 h after seeding, cells were dissociated by trypsinization. A volume of 50 µL of cell suspension was mixed with 50 µL of trypan blue dye and subsequently observed to ascertain whether the cells became colored or not through visual inspection. The fraction of viable cells in 1 mL was calculated using the following formula:

$$\text{Nb of viable cells per 1}\;\mathrm{mL}=\frac{Nb\;of\;counted\;cells\times Dilution\;factor\times 10^{4}}{Nb\;of\;counted\;quadrants}$$

Each experiment was repeated 3 times.

All experiments were performed between passages 4 and 8.

### 2.6. Immunofluorescence

Cells were seeded on 12 mm glass coverslips in 24-well plates. After amplification, cells were irradiated at a dose of 2 Gy, which is a typical radiotherapy session. Irradiated cells were fixed at 0 min (without irradiation), 10 min, 1 h, 4 h and 24 h after the irradiation. Cells were fixed in 4% paraformaldehyde at room temperature for 15–20 min. The PFA was aspirated, and cells were washed twice with PBS (Sigma-Aldrich, St. Louis, MO, USA) at room temperature.

Cells were seeded on 12 mm glass coverslips in 24-well plates. After amplification, cells were fixed in 4% paraformaldehyde at room temperature for 15–20 min. The PFA was aspirated, and cells were washed twice with PBS (Sigma-Aldrich, St. Louis, MO, USA) at room temperature.

Cells were then permeabilized and blocked with a mixture of 0.5% Triton-X100 (Sigma-Aldrich, St. Louis, MO, USA), 10% normal goat serum (NGS-Gibco, Grand Island, NY, USA), and 3% bovine serum albumin (BSA- Sigma-Aldrich, St. Louis, MO, USA) for 1 h at room temperature. Non-irradiated cells were then incubated with anti-E Cadherin anti-mouse antibody (Abcam, Cambridge, UK; cat #ab11512 at a dilution 1/100), and with anti-Vimentin monoclonal anti-mouse antibody (Abcam, Cambridge, UK; cat #ab92547 at a dilution 1/50), for 1 h at 37 °C. After gentle washing with PBS, cells were incubated with secondary antibodies: Alexa Fluor-488 conjugated IgG (Abcam, Cambridge, UK; cat #ab150113 at a dilution 1/100) and Alexa Fluor-568 conjugated IgG (Abcam, Cambridge, UK; cat # ab175471 at a dilution 1/100), respectively, for anti-E Cadherin and anti-Vimentin, for 30 min at 37 °C.

Irradiated and non-irradiated cells were incubated with anti-γH2AX (ser139) anti-mouse antibody (Millipore, Burlington, MA, USA; cat #05636 at a dilution 1/350), and with anti-pATM (ser1981) monoclonal anti-mouse antibody (Millipore, Burlington, MA, USA; cat #05740 at a dilution 1/80), for 1 h at 37 °C. After gentle washing with PBS, cells were incubated with secondary antibodies (Alexa-488 conjugated IgG; Abcam, Cambridge, UK; cat #ab150113 at a dilution 1/100) for 30 min at 37 °C. Cells were then washed, and 20 µL of the anti-fade reagent Fluoro-gel II with DAPI (Abcam, Cambridge, UK; cat #ab104139) was added directly on the slide before mounting the coverslip. Confocal microscopic analyses were performed using a Zeiss LSM 710 laser scanning confocal microscope (Oberkochen, Carl-Zeiss-Straße 22, Germany) and images were acquired and analyzed using the Zeiss LSM image software (ZEN). The number of nuclear foci was scored manually in at least 30 nuclei per condition and per experiment. The percentage of cells with 2 or more micronuclei was assessed with manual DAPI staining for each condition and experiment. Each experiment was repeated at least three times. DAPI staining also permitted the indirect evaluation of the yield of G1 cells (nuclei with homogeneous DAPI staining), G2 cells (nuclei with heterogeneous DAPI staining), and metaphase (visible chromosomes), and the ability to only focus on G0/G1 cells [18]. We applied separated IF in order to avoid any bias due to co-immunofluorescence [19]. All experiments were performed between passages 4 and 8.

### 2.7. Cell Survival Clonogenic Assay

Cells were plated in 6-well culture plates and incubated at 37 °C in a humidified incubator containing 5% CO_2_. Upon reaching 50–60% confluency, cells were irradiated with a single dose of 2 Gy. A clonogenic assay with the delayed plating technique was performed. Twenty-four hours after irradiation, 7000 to 10,000 cells were plated in a T25 culture flask. Cells were kept at 37 °C in a humidified incubator containing 5% CO_2_ for 10–20 days to form colonies. Cells were then fixed with 95% ethanol for 1 min and stained with crystal violet for 3 min, after which they were washed with distilled water twice. Colonies were considered as such if they were composed of more than 50 cells [11,12]. Each experiment was repeated at least 3 times. All experiments were performed between passages 4 and 8.

### 2.8. Statistical Analysis

Statistical analysis was performed using IBM SPSS statistics software (Version 29.0.0.0 (241). The Shapiro–Wilk test was performed to confirm data normality [20]. A one-way ANOVA followed by a Bonferroni post hoc analysis was performed to compare the data of each condition and time-point. Differences were considered statistically significant when *p*-value < 0.05.

## 3. Results

### 3.1. Fibroblastic Nature of the Established Cell Lines

**Figure 2:**
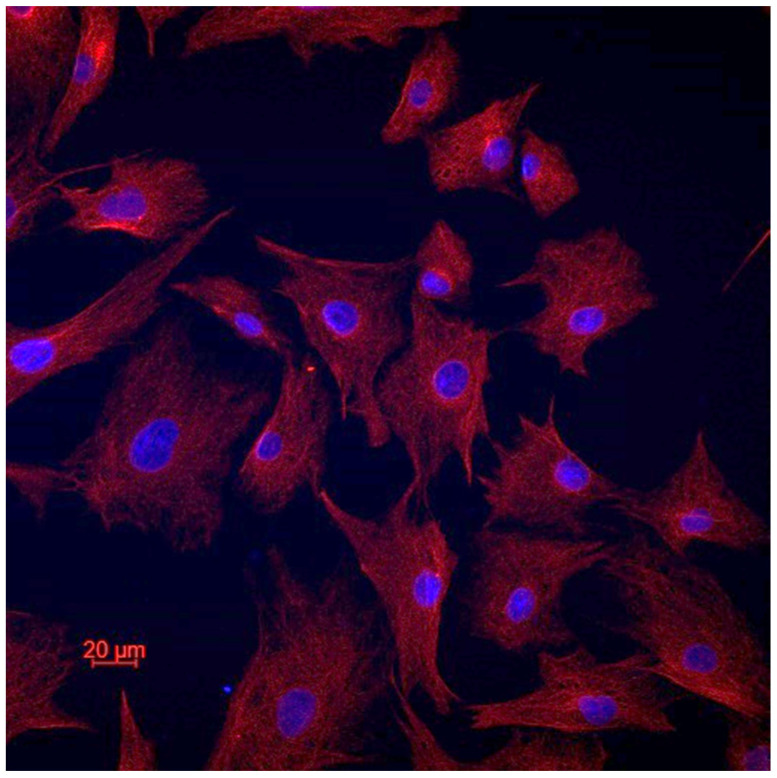
Representative image of vimentin staining.

In order to confirm the cellular type of the established cell lines, we performed vimentin staining (Figure 2). All the cells of all the cell lines expressed vimentin, which confirms their fibroblastic origin [21]. There was no e-cadherin expression in all the cell lines.

### 3.2. Previous Irradiation Does Not Affect Cellular Viability

**Figure 3:**
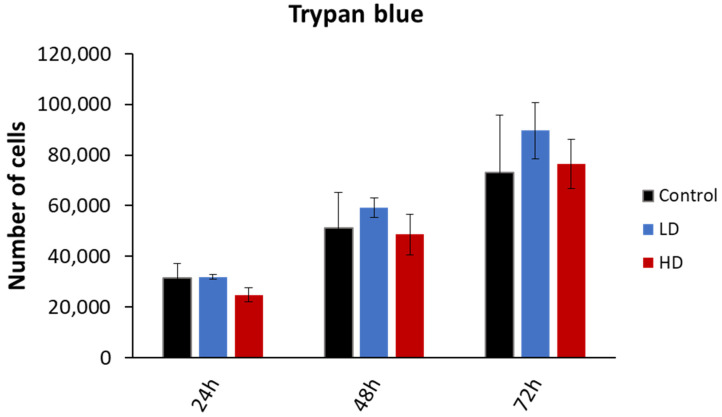
Effect of previous irradiation on cell viability. Results are represented as the average values of 6 control, 4 LD, and 5 HD sheep lung fibroblasts ± the standard error of the mean. A one-way ANOVA was performed to compare the number of cells at different time-points with each other (NS: no significance). Experiments were performed between passages 4 and 8. Results showed that there was no statistically significant difference in the cellular proliferation between the control, LD and HD cells.

The cell viability was assessed through the trypan blue dye exclusion assay. The experiment was performed on 15 cell lines (6 control, 4 LD and 5 HD; Figure 3). While the number of viable cells increased with time, there was no statistically significant difference between the control, LD and HD cell lines (*p* > 0.05). The same was observed when the data were grouped as treated vs. non-treated (*p* > 0.05). The experiments were performed between passages 4 and 8.

### 3.3. Previous Irradiation Impairs DNA DSB Signaling and Repair

**Figure 4:**
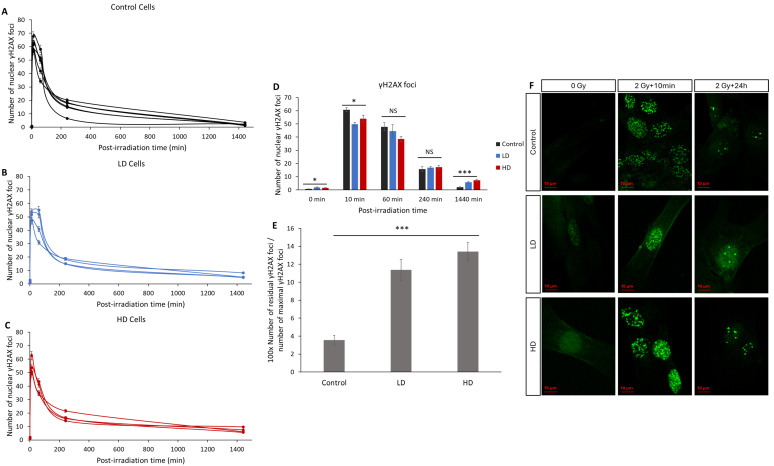
Effect of previous irradiation on the kinetics of γH2AX foci. (**A**) Kinetics of γH2AX in the control cell lines. (**B**) Kinetics of γH2AX in LD cell lines. (**C**) Kinetics of γH2AX in HD cell lines. Cells were irradiated with 2 Gy then fixed at 0 Gy 10 min, 1 h, 4 h, and 24 h after irradiation. For each condition, the number of foci in at least 30 nuclei was counted, and each experiment was repeated 3 times. (**D**) Average number of foci for control, LH and HD cell lines. Results are shown as the mean ± standard error of the mean. A one-way ANOVA test followed by a Bonferroni post hoc analysis was performed to compare the average number of foci at each time-point for the three groups of lung fibroblasts (*: *p* < 0.05; ***: *p* < 0.001; NS: no significance. Analysis showed that both LD and HD cells had a higher number of basal γH2AX foci (*p* < 0.05), residual γH2AX foci (*p* < 0.001) and a lower number of foci per cell 10 min post-irradiation (*p* < 0.05) when compared with the control cells. (**E**) Ratio of the number of residual foci to the number of maximal foci. (**F**) Representative images of anti-γH2AX staining. Experiments were performed between passages 4 and 8.

The number of radio-induced γH2AX foci is the main biomarker for DNA DSBs and it is widely used to characterize the intrinsic radiosensitivity [9,11,22,23]. Here, we assessed the kinetics of the appearance and disappearance of nuclear γH2AX foci on 15 cell lines after 2 Gy of irradiation (Figure 4). Without irradiation, the number of spontaneous foci in the LD and HD cell lines was slightly but significantly higher than that in the control lines (0.6 ± 0.12 for the control vs. 1.8 ± 0.41 and 1.5 ± 0.5 foci for LD and HD cell lines, respectively; *p* < 0.05). After irradiation, the number of foci in all the cell lines followed the typical shape associated with DNA damage signaling and repair, with a sharp increase followed by a decrease over 24 h [24]. A duration of 10 min after exposure to 2 Gy of irradiation, the number of foci increased significantly and was significantly higher in the control cell lines (60 ± 1.6 foci) when compared with the LD (50 ± 1.4 foci) and HD (54 ± 2.6 foci) cell lines (*p* < 0.05). However, the most significant difference was observed in the residual number of foci 24 h post-irradiation: while only 2.1 ± 0.3 foci were counted, on average, in the control cells, the LD and HD cells had a remaining 5.7 ± 0.7 and 7.25 ± 0.8 foci (*p* < 0.001). When grouped as the control vs. treated cell lines, the differences were even more significant (*p* < 0.001). In order to better understand the effect of the previous treatment on the kinetics of the appearance and disappearance of γH2AX foci, the ratio of the number of residual foci 24 h post-RT to the number of foci 10 min post-RT was calculated (Figure 4E). The difference between the control cells and LD and HD cells was even more pronounced (*p* < 0.001). Here, also, there was no statistically significant difference between the LH and HD cells (*p* > 0.05).

### 3.4. ATM Nucleoshuttling Is Delayed in Previously Treated Cells

**Figure 5:**
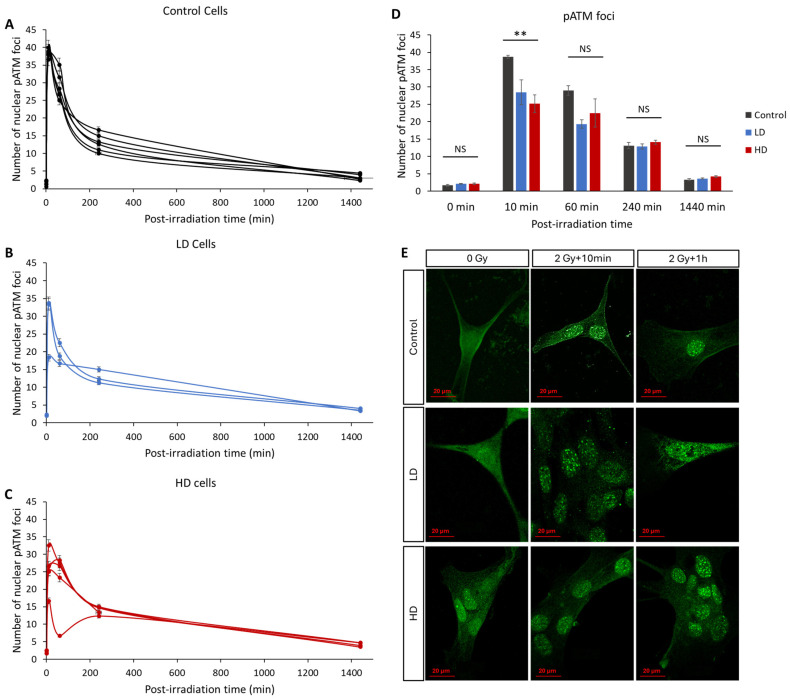
Effect of a previous irradiation on the kinetics of pATM foci. (**A**) Kinetics of pATM in the control cell lines. (**B**) Kinetics of pATM in LD cell lines. (**C**) Kinetics of pATM in HD cell lines. Cells were irradiated with 2 Gy then fixed at 0 min (without irradiation) 10 min, 1 h, 4 h, and 24 h after irradiation. For each condition, the number of foci in at least 30 nuclei was counted, and each experiment was repeated 3 times. Error bars are not shown for clarity. (**D**) Average number of pATM foci for control, LH and HD cell lines. Results are shown as the mean ± standard error of the mean. A one-way ANOVA test followed by a Bonferroni post hoc analysis was performed to compare the average number of foci at each time-point for the three groups of lung fibroblasts (**: *p* < 0.01; NS: no significance). Analysis showed that LD and HD cells exhibited a lower number of pATM foci 10 min post-irradiation (*p* < 0.01) when compared with the control cells. (**E**) Representative images of anti-pATM staining. All experiments were performed between passages 4 and 8.

In recent years, many studies have confirmed that the kinetics of the radio-induced nucleoshuttling of the ATM protein can accurately predict and describe the molecular, cellular and clinical radiosensitivity [9,10,11,12,13,25]. In our study, we exposed the control, LD and HD cell lines to a 2 Gy irradiation dose, and the kinetics of the appearance and disappearance of pATM foci was assessed (Figure 5). Without irradiation, there was no statistically significant difference in the basal number of foci between the three groups (*p* > 0.05). The maximal value was reached either 10 min or 1 h after irradiation, depending on the cell line. However, 10 min after irradiation, the LD and HD cells had a significantly lower number of pATM foci (38 ± 0.5 foci for the control cells vs. 28 ± 3.5 and 25 ± 2.5 for LD and HD cell lines). This difference was even more significant when the previously treated cells were grouped (*p* < 0.01). For all the other time-points, there was no difference between the control and the treated groups. It is noteworthy to mention that there was no statistically significant difference between the LD and HD cell lines for all the conditions.

### 3.5. Previous Irradiation Did Not Affect the Percentage of Radio-Induced Micronuclei or Cell Survival

The percentage of cells with micronuclei can be correlated with radiosensitivity and chromosomal aberrations [26,27,28]. After treating the cells with 2 Gy of irradiation, we measured the percentage of cells with micronuclei without irradiation 10 min, 1 h, 4 h and 24 h after treatment (Figure 5). No significant difference was observed between the control, LD and HD cell lines for all time-points (*p* > 0.05). When combining the LD and HD values, there was no significant difference between the treated and the control cells (*p* > 0.05).

**Figure 6:**
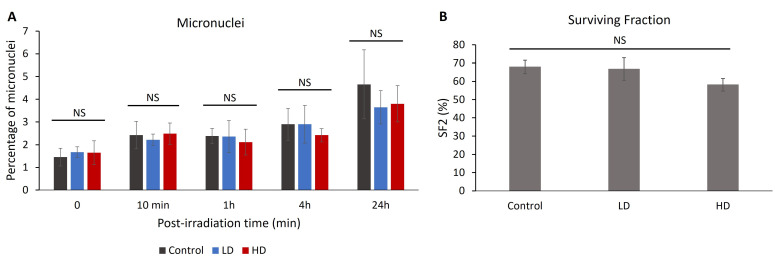
(**A**) Effect of previous irradiation on the percentage of micronuclei and cell survival. For each cell line, the percentage of cells with more than 2 micronuclei was assessed in 3 independent experiments. Results show that there is no statistically significant difference in the percentage of micronuclei between the control, LD and HD cells. (**B**) Effect of previous irradiation on cellular radiosensitivity. A total of 6 control, 4 LD and 5 LD cell lines were treated with a single dose of 2 Gy of irradiation, and the clonogenic assay was performed. Data show that there were no statistically significant differences between the SF2 values of the control, LD and HD cells. Results are represented as the average values of 6 control, 4 LD and 5 LD sheep lung fibroblasts ± the standard error of the mean. A one-way ANOVA was performed to compare the number of cells at different time-points with each other (NS: no significance). Experiments were performed between passages 4 and 8.

Since 1956, the clonogenic assay has been the main cellular radiosensitivity test used in radiobiology [29]. The capacity of the irradiated cells to form colonies is inversely correlated with the clinical response to radiation [30,31,32]. A total of 15 cell lines (6 control, 4 LD and 5 HD) received 2 Gy of irradiation and the clonogenic assay with the delayed plating technique was performed (Figure 6). The value of the surviving fraction after 2 Gy of irradiation (SF2) ranged between 46% and 80%. However, there was no statistically significant difference between the control, LD and HD cell lines, showing that previous irradiation did not have any effect on the capacity of the cells to form colonies after 2 Gy of irradiation.

## 4. Discussion

### 4.1. Pediatric Radiotherapy: Long-Term Consequences

Tumors of the nervous system are the most common solid tumors in pediatric cancers, with medulloblastoma (MB) being the most diagnosed pathology [1]. MB is usually treated by craniospinal irradiation after surgical resection [2]. Recent developments in cancer treatments have increased the survival rates of childhood cancer patients; however, it is well established that in the long term, these survivors are at a higher risk of developing secondary cancers or having other types of medical diseases [33,34,35,36]. This can be caused by many factors, including their genetic predisposition to cancer, in addition to early exposure to high doses of RT and CT [3,37,38,39]. Moreover, when performing CSI, different organs in the irradiation field receive a non-negligible dose. This can lead to long-term complications with no clear indication on their behavior at the molecular and cellular level, especially whenever re-exposed to genotoxic stress.

While the clinical evidence concerning the long-term toxicities of RT is abundant, there are no studies that focus on the early irradiation effects on the molecular and cellular radiosensitivity and cancer predisposition. Here, our choice of animal model was motivated by different considerations:

- Animal size: This study required an animal model the size of a very young human, in order to have the most relevant treatment plan. It is noteworthy that the bone structure and composition of sheep are very similar to those of the human body [40].
- Life expectancy: This study required an animal model with a life expectancy long enough to observe any effects, but not too long for technical considerations. The life expectancy of sheep ranges between 8 and 10 years, which made them ideal for this project.
- Adult age: This study required an animal that reaches adulthood at ages 2–4 years in order to assess the effect of early pediatric treatment at adulthood.
- Radiosensitivity and DNA repair: The non-homologous end-joining and the homologous recombination repair pathways, which involved ATM and H2AX, are assumed to be the two DSB repair pathways present in mammalian cells [41,42]. Specifically, γH2AX is detected in all tested mammalian cells [43,44,45]. This justifies our focus on these two proteins. Moreover, as highlighted in [15], and as confirmed in our results, the cellular and molecular radiosensitivity of sheep was shown to be comparable to that of humans.

In conclusion, even though we cannot directly correlate our results in ovine models with the potential effects in humans, we believe that this was the most relevant model that might describe the long-term effects of pediatric radiotherapy on lung tissues.

In our study, we treated two groups of sheep with a treatment equivalent to the one performed for pediatric central nervous system (CNS) tumors. It is noteworthy that all the sheep included in this study seemed healthy with no specific health issues. Moreover, no signs of tumor development were observed at the end of this study. However, this does not contradict our findings, since we know that (1) cancer is a multifactorial disease, and an increase in cancer risk does not mean that a tumor will eventually develop and (2) the experiment ended when the sheep were approximately 4 years old. With the sheep life expectancy ranging between 10 and 12 years, we cannot exclude the fact that, if kept alive, some animals could have developed a tumor at a later stage in their life.

### 4.2. Previous Irradiation Can Radiosensitize Cells by Impairing DNA DSB Signaling and Repair

The intrinsic radiosensitivity is generally correlated with the capacity of the cells to repair induced DNA DSBs. Extensive data have shown that a dose of 1 Gy of X-rays can induce between 35 and 40 DNA DSBs [23,24]. In parallel, irradiation activates DNA DSB signaling and repair pathways, which starts by the monomerization of the cytoplasmic ATM dimers, followed by ATM nucleoshuttling, or the transit of ATM monomers from the cytoplasm to the nucleus. Once in the nucleus, ATM will phosphorylate the H2AX histone variant at the DSB site, which will trigger a cascade of phosphorylation events that will eventually lead to the repair of the detected DSBs [4]. Recent data have shown that any decrease or delay in the ATM activity can lead to a higher risk of radiation-induced complications [11]. These studies have defined three radiosensitivity groups [9]:

- Group I: radioresistant. Cells from this group show efficient DNA DSB repair and fast ATM nucleoshuttling. The usual number of residual γH2AX foci in this group ranges between 0 and 2, and the number of pATM foci at 10 min is higher than 35.
- Group II: moderately radiosensitive. Cells show less efficient DNA DSB repair and slower ATM nucleoshuttling. These can be radiosensitive and/or with high cancer proneness. The number of residual γH2AX foci is between 2 and 8 and the number of pATM foci is between 25 and 35.
- Group III: hyper-radiosensitive. These usually include DNA repair genetic mutation, such as that present in ataxia telangiectasia. This group has a high risk of cancer and more than eight residual γH2AX foci.

In our results, we showed that all the cell lines provided from previously irradiated sheep reveal the following:

1. A small but significant increase in the number of basal DNA DSBs was detected, as highlighted by the number of γH2AX foci without irradiation (Figure 3). Although the increase was small, the significant difference shows that the previously irradiated cells can continuously have remaining DNA DSBs, even without any exposure to genotoxic stress. Many factors contribute to this phenomenon, for example, the unrepaired DSBs from environmental stress, or an increase in the activity of reactive oxygen species. Studies have shown that this can also be a sign of cell aging [46].
2. A significant decrease in the number of recognized DSBs, highlighted by the number of γH2AX foci 10 min after irradiation. Knowing that the number of radio-induced DSBs should be the same, this shows that LD and HD cells might have a DSB signaling problem.
3. A significant decrease in the ATM activity 10 min after irradiation. Knowing that ATM acts early after exposure to genotoxic stress, multiple studies have shown that any decrease or delay in ATM activity can lead to genomic instability and increased radiosensitivity.
4. A significant increase in the number of residual γH2AX foci 24 h after irradiation. This means that previous irradiation might have affected the capacity of the treated cells to repair their DNA DSBs upon re-irradiation.

While most radiation research studies assume that the number of residual γH2AX foci is correlated with the number of unrepaired DSB, some reports have shown that this remaining phosphorylation can be caused by other mechanisms [47,48,49,50,51,52].

However, even though the exact definition of residual foci might be debatable, the correlation between the residual number of γH2AX foci and molecular, cellular and clinical radiosensitivity is very well documented [48,51,53,54,55,56,57,58,59,60,61,62]. The sensitivity and repeatability of the γH2AX assay also makes it one of the most reliable biomarkers for unrepaired DSBs [63].

Overall, the control cells were in Group I regarding their radiosensitivity, while the LD and HD cells were in the group II. While it might be argued that the difference in the DNA repair capabilities might have been caused by genetic and intrinsic radiosensitivity factors, the big difference in both the pATM and γH2AX values at different time-points after the irradiation suggests that this explanation is unlikely, and that the main cause remains the previous RT.

Previous irradiation might have caused this transition between both groups by modifying the activity of DNA DSB repair proteins and/or by inducing mutations in genes coding for these proteins. These results were consistent in all 15 tested primary cell lines. Moreover, our results show one of the few quantifications of the long-term effect of irradiation on DSB repair kinetics.

Interestingly, there was no difference between the LD and HD cell lines in all the performed assays. Our interpretation of this is that previous irradiation causes impairment of the DNA DSB repair pathways that is not dose-dependent. This can hypothetically be explained by the assumption that most of the cells that received the high dose in the HD region did not survive the initial treatment. However, the minority of the most resistant cells that were able to survive displayed the same effect as if they were treated with a lower dose.

We found no statistically significant difference in the percentage of MN and SF2. This indicates that the differences in the DSB repair capabilities were not enough to increase the cellular radiosensitivity with a 2 Gy irradiation dose. More experiments with different doses and dose fractionations would confirm the effect of previous irradiation on cellular radiosensitivity at higher doses.

### 4.3. Clinical Relevance

The role of DNA damage repair proteins is to protect the integrity of our genome, and limit mutations that can lead to the development of cancer. A decrease in the capacity of the cells to repair DNA damage will increase the risk of radio-induced toxicities and cancer [64]. In our study, we tried to mimic the effect of a pediatric radiotherapy treatment on sheep as models. We showed that previous irradiation at a very young age impaired the capacity of the cells to recognize and repair DNA DSBs. This means that, whenever the target is exposed to any genotoxic stress, such as chemicals, radiation, pollution, tobacco, etc., the risk of unrepaired and/or misrepaired DSBs increases significantly when the subject received pediatric exposure to ionizing radiation [65,66,67]. This, in turn, will increase the risk of mutations, and these mutations can target oncogenes and/or tumor suppressor genes, leading therefore to the development of cancer [68]. The fact that pediatric cancer survivors have a relatively long life expectancy due to improved treatments and their young age, further increases the importance of mitigating their risk of exposure to such damaging agents [69]. Moreover, RT might modify childhood cancer survivors’ DNA DSB repair capabilities, increasing their radiosensitivity. This will increase their risk of radiation-induced toxicities if another RT treatment was to be performed. Remaining to be analyzed is the effect of other particles, such as protons or carbon ions. Testing this could be of great importance because if other means of irradiation show decreased damage, the benefit will add up to the already proven dosimetric sparing of normal tissue with these novel particles.

There were limiting factors to our study, such as the animal models used. Still, other animal models, such as pigs, should be considered for a follow-up study. Our conclusions should also be confirmed by other studies because of the relatively small number of animals included in this study. Moreover, the effect of sedation with ketamine on the radioresponse of tissues should also be investigated.

The effect of early RT on other tissues, in addition to irradiation with different doses, with or without radiomodulating drugs, should also be assessed [70,71,72]. Additional DNA damage response biomarkers should also be investigated, such as CHK2, CHK1, p53 and 53BP1. More molecular assays such as Western blotting could also confirm our results. Finally, senescence biomarkers and other cell death assays should be performed with and without irradiation at different doses.

## 5. Conclusions

In conclusion, in our study, we quantified the long-term effect of pediatric RT on the radiosensitivity of lung fibroblasts in ovine models. We showed that exposure to RT at a young age can decrease the capacity of lung fibroblasts to repair DNA DSBs, which can lead to an increase in the risk of secondary malignancies and radiosensitivity to other late detriments.

## Data Availability

Data can be shared upon request from the corresponding author.
